# Jacobi spectral collocation method for the approximate solution of multidimensional nonlinear Volterra integral equation

**DOI:** 10.1186/s40064-016-3358-z

**Published:** 2016-10-04

**Authors:** Yunxia Wei, Yanping Chen, Xiulian Shi, Yuanyuan Zhang

**Affiliations:** 1 College of Mathematic and Information Science, Shandong Institute of Business and Technology, Yantai, 264005 China; 2 School of Mathematical Sciences, South China Normal University, Guangzhou, 510631 China; 3 School of Mathematics and Statistics, Zhaoqing University, Zhaoqing, 526061 China; 4 Department of Mathematics and Information Science, Yantai University, Yantai, 264005 China

**Keywords:** Multidimensional nonlinear Volterra integral equation, Jacobi collocation discretization, Multidimensional Gauss quadrature formula, Error estimates, 65R20, 45J05, 65N12

## Abstract

We present in this paper the convergence properties of Jacobi spectral collocation method when used to approximate the solution of multidimensional nonlinear Volterra integral equation. The solution is sufficiently smooth while the source function and the kernel function are smooth. We choose the Jacobi–Gauss points associated with the multidimensional Jacobi weight function $\omega ({\mathbf{x}})=\Pi _{i=1}^d(1-x_i)^\alpha (1+x_i)^\beta ,\; -1<\alpha , \beta <\frac{1}{d}-\frac{1}{2}$ (*d* denotes the space dimensions) as the collocation points. The error analysis in $L^\infty$-norm and $L_\omega ^2$-norm theoretically justifies the exponential convergence of spectral collocation method in multidimensional space. We give two numerical examples in order to illustrate the validity of the proposed Jacobi spectral collocation method.

## Background

We observe that there are many numerical approaches for solving one-dimensional Volterra integral equation, such as Runge–Kutta method (Brunner 1984; Yuan and Tang 1990), polynomial collocation method (Brunner 1986; Brunner et al. 2001; Brunner and Tang 1989), multistep method (Mckee 1979; Houwen and Riele 1985), hp-discontinuous Galerkin method (Brunner and Schötzau 2006) and Taylor series method (Goldfine 1977). The spectral collocation method is the most popular form of the spectral methods among practitioners. It is convenient to implement for one-dimensional problems and generally leads to satisfactory results an long as the problems possess sufficient smoothness. In the literature (Tang et al. 2008), the authors proposed a Legendre spectral collocation method for Volterra integral equation with a regular kernel in one-dimensional space. Subsequently, Chen and Tang (2009, 2010), Chen et al. (2013), developed the spectral collocation method for one-dimensional weakly singular Volterra integral equation. The proofs of the convergence properties of spectral collocation method for Volterra integro-differential equation with a single spatial variable are given in Wei and Chen (2012a, b, 2013, 2014). Nevertheless, to the best of our knowledge, there have been no works regarding the theoretical analysis of the spectral approximation for multidimensional Volterra integral equation (Atdev and Ashirov 1977; Beesack 1985; Pachpatte 2011; Suryanarayana 1972), even for the case with smooth kernel.

We shall extend to several space dimensions the approximation results in Tang et al. (2008) for a single spatial variable. The expansion of Jacobi will be considered. We will be concerned with Sobolev-type norms that are most frequently applied to the convergence analysis of spectral methods. We get the discrete scheme by using multidimensional Gauss quadrature formula for the integral term. We will provide a rigorous verification of the exponential decay of the errors for approximate solution.

We study the multidimensional nonlinear Volterra integral equation of the form

1 $$\begin{aligned} & y(t_1,t_2,\ldots ,t_d)+\int _0^{t_1}\int _0^{t_2}\cdots \int _0^{t_d}K(t_1,s_1,t_2,s_2,\ldots ,t_d,s_d,y(s_1,s_2,\ldots ,s_d)) \\&\quad ds_d\ldots ds_2ds_1=g(t_1,t_2,\ldots ,t_d),\quad t_i\in [0,T_i],\quad i=1,2,\ldots ,d, \end{aligned}$$

by the Jacobi spectral collocation method. Here, $g: [0,T_1]\times [0,T_2]\times \cdots \times [0,T_d] \rightarrow R$ and $K: D\times R\rightarrow R$ (where $D:=\{(t_1,s_1,t_2,s_2,\ldots ,t_d,s_d): 0\le s_i\le t_i\le T_i, i=1,2,\ldots ,d$}) are given smooth functions. If the given functions are smooth on their respective domains, the solution *y* is also the smooth function (see Brunner 2004). This fact will be the standing point of this paper.

## Discretization scheme

We consider now the domain $\Omega =(-1,1)^d$ and we denote an element of ${\mathbb {R}}^d$ by ${\mathbf{x}}=(x_1,x_2,\ldots ,x_d)$. Let $-1<\alpha , \beta <\frac{1}{d}-\frac{1}{2}$, if $\omega =\omega ({\mathbf{x}})=\Pi _{i=1}^d(1-x_i)^\alpha (1+x_i)^\beta$ denotes a d-dimensional Jacobi weight function on $\Omega$, we denote by $L_\omega ^2(\Omega )$ the space of the measurable functions $u:\Omega \rightarrow {\mathbb {R}}$ such that $\int _\Omega |u({\mathbf{x}})|^2\omega ({\mathbf{x}})d{\mathbf{x}}<+\infty$. It is a Banach space for the norm

$$\Vert u\Vert _{L_\omega ^2(\Omega )}=\left( \int _\Omega |u({\mathbf{x}})|^2\omega ({\mathbf{x}}) d{\mathbf{x}}\right) ^{\frac{1}{2}}.$$

The space $L_\omega ^2(\Omega )$ is a Hilbert space for the inner product

$$(u,v)_\omega =\int _\Omega u({\mathbf{x}})v({\mathbf{x}})\omega ({\mathbf{x}})d{\mathbf{x}}.$$

$L^\infty (\Omega )$ is the Banach space of the measurable functions $u:\Omega \rightarrow {\mathbb {R}}$ that are bounded outside a set of measure zero, equipped with the norm

$$\Vert u\Vert _{L^\infty (\Omega )}=ess\;sup_{{\mathbf{x}}\in \Omega }|u({\mathbf{x}})|.$$

Given a multi-index $\alpha =(\alpha _1,\alpha _2,\ldots ,\alpha _d)$ of nonnegative integers, we set

$$|\alpha |=\alpha _1+\alpha _2+\cdots +\alpha _d$$

and

$$D^\alpha v=\frac{\partial ^{|\alpha |}v}{\partial _{x_1}^{\alpha _1}\partial _{x_2}^{\alpha _2}\cdots \partial _{x_d}^{\alpha _d}}.$$

We define $H_\omega ^m(\Omega )$= {$v\in L_\omega ^2(\Omega )$:   for each nonnegative multi-index $\alpha$ with $|\alpha |\le m$, the distributional derivative $D^\alpha v$ belongs to $L_\omega ^2(\Omega )\}.$ This is a Hilbert space for the inner product

$$(u,v)_{m,\omega }=\sum _{|\alpha |\le m}\int _\Omega D^\alpha u({\mathbf{x}})D^\alpha v({\mathbf{x}})\omega ({\mathbf{x}})d{\mathbf{x}},$$

which induces the norm

$$\Vert v\Vert _{H_\omega ^m(\Omega )}=\left( \sum _{|\alpha |\le m}\Vert D^\alpha v\Vert ^2_{L_\omega ^2(\Omega )}\right) ^{\frac{1}{2}}.$$

Let $\{\tilde{x}_j, 0\le j\le N\}$ denote the Jacobi Gauss points on the one-dimensional interval $(-1,1)$ (see Canuto et al. 2006; Shen and Tang 2006). We now consider multidimensional Jacobi interpolation. Let ${\mathbb {P}}_N(\Omega )$ be the space of all algebraic polynomials of degree up to *N* in each variable $x_i$ for $i=1,2,\ldots ,d$. Let us introduce the Jacobi Gauss points in $\Omega$:

$$\tilde{{\mathbf{x}}}_{\mathbf{j}}=({\tilde{x}}_{j_1},{\tilde{x}}_{j_2},\ldots ,{\tilde{x}}_{j_d}) \text { for }{\mathbf{j}}=(j_1,j_2,\ldots ,j_d)\in \mathbb {N}^d,\quad |{\mathbf{j}}\Vert =\max \limits _{1\le i\le d}j_i\le N,$$

and denote by $I_N$ the interpolation operator at these points, i.e., for each continuous function *u*, $I_Nu\in {\mathbb {P}}_N$ satisfies

$$(I_Nu)(\tilde{{\mathbf{x}}}_{\mathbf{j}})=u(\tilde{{\mathbf{x}}}_{\mathbf{j}})\text { for all }{\mathbf{j}}\in \mathbb {N}^d,\quad |{\mathbf{j}}\Vert \le N.$$

We can represent $I_Nu$ as follows:

$$I_Nu({\mathbf{x}})=\sum _{\Vert {\mathbf{j}}\Vert \le N}u(\tilde{{\mathbf{x}}}_{\mathbf{j}})F_{\mathbf{j}}({\mathbf{x}}),$$

where $F_{\mathbf{j}}({\mathbf{x}})=F_{j_1}(x_1)F_{j_2}(x_2)\ldots F_{j_d}(x_d)$, $\{F_j\}_{j=0}^N$ is the Lagrange interpolation basis function associated with the Jacobi collocation points $\{\tilde{x}_j\}_{j=0}^N$. The multidimensional Jacobi Gauss quadrature formula is

2 $$\begin{aligned} & \int _\Omega f({\mathbf{x}})d{\mathbf{x}}\approx \sum _{\Vert {\mathbf{j}}\Vert \le N} f({\tilde{x}}_{j_1},{\tilde{x}}_{j_2},\ldots ,{\tilde{x}}_{j_d})\omega _{j_1}\omega _{j_2}\ldots \omega _{j_d}. \end{aligned}$$

We use the variable transformations $t_i=\frac{T_i}{2}(1+x_i),\; x_i\in [-1,1]$ and $s_i=\frac{T_i}{2}(1+\tau _i),\; \tau _i\in [-1,x_i],\;i=1,2,\ldots ,d$ to rewrite (1) as follows

3 $$u(x_1,x_2,\ldots ,x_d)+\int _{-1}^{x_1}\int _{-1}^{x_2}\cdots \int _{-1}^{x_d}{\hat{K}}(x_1,\tau _1,x_2,\tau _2,\ldots ,x_d,\tau _d, u(\tau _1, \tau _2,\ldots ,\tau _d))d\tau _d\ldots d\tau _2d\tau _1 =f(x_1,x_2,\ldots ,x_d).$$

Here,

$$f(x_1,x_2,\ldots ,x_d)=g\left( \frac{T_1}{2}(1+x_1), \frac{T_2}{2}(1+x_2),\ldots ,\frac{T_d}{2}(1+x_d)\right) , {\hat{K}} (x_1,\tau _1,x_2,\tau _2,\ldots ,x_d,\tau _d,u)= \frac{T_1}{2}\frac{T_2}{2}\cdots \frac{T_d}{2} K\left( \frac{T_1}{2}(1+x_1),\frac{T_1}{2}(1+\tau _1),\frac{T_2}{2}(1+x_2), \frac{T_2}{2}(1+\tau _2),\ldots ,\frac{T_d}{2}(1+x_d),\frac{T_d}{2}(1+\tau _d),u\right),$$

and $u(x_1,x_2,\ldots ,x_d)=y\left( \frac{T_1}{2}(1+x_1),\frac{T_2}{2}(1+x_2), \ldots ,\frac{T_d}{2}(1+x_d)\right)$ is the smooth solution of problem (3).

Firstly, Eq. (3) holds at the collocation points $\tilde{{\mathbf{x}}}_{\mathbf{j}}=({\tilde{x}}_{j_1},{\tilde{x}}_{j_2}, \ldots ,{\tilde{x}}_{j_d})$ on $\Omega$, i.e.,

4 $$u({\tilde{x}}_{j_1},{\tilde{x}}_{j_2},\ldots ,{\tilde{x}}_{j_d})+ \int _{-1}^{{\tilde{x}}_{j_1}} \int _{-1}^{\tilde{x}_{j_2}}\cdots \int _{-1}^{\tilde{x}_{j_d}}{\hat{K}}({\tilde{x}}_{j_1},\tau _1, {\tilde{x}}_{j_2},\tau _2,\ldots ,{\tilde{x}}_{j_d}, \tau _d, u(\tau _1,\tau _2,\ldots ,\tau _d))d\tau _d\cdots d\tau _2d\tau _1=f({\tilde{x}}_{j_1},{\tilde{x}}_{j_2},\ldots ,{\tilde{x}}_{j_d}).$$

In order to obtain high order accuracy for the problem (4), we transfer the integral domain $[-1,{\tilde{x}}_{j_1}]\times [-1,{\tilde{x}}_{j_2}]\cdots \times [-1,{\tilde{x}}_{j_d}]$ to a fixed interval $\bar{\Omega }$

5 $$u({\tilde{x}}_{j_1},{\tilde{x}}_{j_2},\ldots ,{\tilde{x}}_{j_d}) +\int _{-1}^{1} \int _{-1}^{1}\cdots \int _{-1}^{1}\tilde{K}({\tilde{x}}_{j_1},\tau _1({\tilde{x}}_{j_1},\theta _1), {\tilde{x}}_{j_2}, \tau _2({\tilde{x}}_{j_2},\theta _2),\ldots ,{\tilde{x}}_{j_d}, \tau _d({\tilde{x}}_{j_d},\theta _d), u(\tau _1({\tilde{x}}_{j_1},\theta _1), \tau _2({\tilde{x}}_{j_2},\theta _2),\ldots ,\tau _d({\tilde{x}}_{j_d},\theta _d)))d\theta _d\cdots d\theta _2d\theta _1 =f({\tilde{x}}_{j_1},{\tilde{x}}_{j_2},\ldots ,{\tilde{x}}_{j_d}),$$

by using the following transformation

6 $$\tau _i=\tau _i({\tilde{x}}_{j_i},\theta _i)=\frac{1+{\tilde{x}}_{j_i}}{2}\theta _i+ \frac{{\tilde{x}}_{j_i}-1}{2},\quad i=1,2,\ldots ,d,$$

where

$$\tilde{K}({\tilde{x}}_{j_1},\tau _1,{\tilde{x}}_{j_2}, \tau _2,\ldots ,{\tilde{x}}_{j_d}, \tau _d,u)= \frac{1+{\tilde{x}}_{j_1}}{2} \frac{1+{\tilde{x}}_{j_2}}{2}\cdots \frac{1+{\tilde{x}}_{j_d}}{2} \hat{K}({\tilde{x}}_{j_1},\tau _1,{\tilde{x}}_{j_2}, \tau _2,\ldots ,{\tilde{x}}_{j_d}, \tau _d,u).$$

Next, let $u_{j_1j_2\cdots j_d}$ be the approximation of the function value $u(\tilde{{\mathbf{x}}}_{\mathbf{j}})$ and use Legendre Gauss quadrature formula, (5) becomes

7 $$\begin{aligned} & u_{j_1j_2\cdots j_d}+\sum _{\Vert {\mathbf{k}}\Vert \le N}\tilde{K}({\tilde{x}}_{j_1},\tau _1({\tilde{x}}_{j_1}, \theta _{k_1}),{\tilde{x}}_{j_2}, \tau _2({\tilde{x}}_{j_2},\theta _{k_2}),\ldots ,{\tilde{x}}_{j_d}, \tau _d({\tilde{x}}_{j_d},\theta _{k_d}), \\& \quad u(\tau _1({\tilde{x}}_{j_1},\theta _{k_1}), \tau _2({\tilde{x}}_{j_2},\theta _{k_2}),\ldots , \tau _d({\tilde{x}}_{j_d},\theta _{k_d}))) \omega _{k_1}\omega _{k_2}\ldots \omega _{k_d} =f({\tilde{x}}_{j_1},{\tilde{x}}_{j_2},\ldots ,{\tilde{x}}_{j_d}). \end{aligned}$$

Here, $\{\theta_{\mathbf{k}}, \Vert {\mathbf{k}}\Vert \le N\}$ denotes the Legendre Gauss points on the multidimensional space $\Omega$ and $\{{\omega }_{\mathbf{k}}, \Vert {\mathbf{k}}\Vert \le N\}$ denotes the corresponding weights. Let $u_N(x_1,x_2,\ldots ,x_d)=\sum\nolimits _{\Vert {\mathbf{i}}\Vert \le N} u_{i_1i_2\ldots i_d}F_{i_1}(x_1)F_{i_2}(x_2)\ldots F_{i_d}(x_d)$. Now, we use $u_N$ to approximate the solution *u*. Then, the Jacobi spectral collocation method is to seek $u_N$ such that $u_{i_1i_2\cdots i_d}$ satisfy the following collocation equation:

8 $$\begin{aligned} & u_{j_1j_2\cdots j_d}+ \sum _{\Vert {\mathbf{k}}\Vert \le N}\tilde{K}({\tilde{x}}_{j_1},\tau _1({\tilde{x}}_{j_1},\theta _{k_1}), {\tilde{x}}_{j_2}, \tau _2({\tilde{x}}_{j_2},\theta _{k_2}),\ldots ,{\tilde{x}}_{j_d}, \tau _d({\tilde{x}}_{j_d},\theta _{k_d}), \\&\quad \sum _{\Vert {\mathbf{i}}\Vert \le N}u_{i_1i_2\ldots i_d}F_{i_1}(\tau _1({\tilde{x}}_{j_1},\theta _{k_1}))F_{i_2} (\tau _2({\tilde{x}}_{j_2},\theta _{k_2}))\ldots F_{i_d}(\tau _d({\tilde{x}}_{j_d},\theta _{k_d}))) \omega _{k_1}\omega _{k_2}\ldots \omega _{k_d} \\&\quad =f({\tilde{x}}_{j_1},{\tilde{x}}_{j_2},\ldots ,{\tilde{x}}_{j_d}). \end{aligned}$$

We can get the values of $u_{i_1i_2\cdots i_d}$ by solving (8) and obtain the expressions of $u_N({\mathbf{x}})$ accordingly.

Let the error function of the solution be written as $e_u({\mathbf{x}}):=u({\mathbf{x}})-u_N({\mathbf{x}})$. Since the exact solution of the problem (1) can be written as $y({\mathbf{t}})=u({\mathbf{x}})\; (t_i=\frac{T_i}{2}(1+x_i),\;t_i\in [0,T_i],\;x_i\in [-1,1])$, we can define its approximate solution $y_N({\mathbf{t}})=u_N({\mathbf{x}})$. Then the corresponding error function satisfy

$$\begin{aligned} & \varepsilon _y({\mathbf{t}}):=y({\mathbf{t}})-y_N({\mathbf{t}}) =e_u({\mathbf{x}})=e_u\left( \frac{2}{T_1}t_1-1,\frac{2}{T_2}t_2-1, \ldots ,\frac{2}{T_d}t_d-1\right) . \end{aligned}$$

### *Remark*

In our work, we let the multidimensional Jacobi weight function $\omega ({\mathbf{x}})=\Pi _{i=1}^d(1-x_i)^\alpha (1+x_i)^\beta ,\; -1<\alpha , \beta <\frac{1}{d}-\frac{1}{2}$. So $\omega (x)=(1-x)^\alpha (1+x)^\beta ,\; -1<\alpha , \beta <\frac{1}{2}$ for $d=1$. In Tang et al. (2008), the authors choose $\alpha =\beta =0$.

## Some lemmas

The following result can be found in Canuto et al. (2006).

### **Lemma 1**

*Assume that Gauss quadrature formula is used to integrate the product* $u\phi$, *where* $u\in H^m(\Omega )$ *for some* $m> \frac{d}{2}$ *and* $\phi \in {\mathbb {P}}_N(\Omega )$. *Then there exists a constant* *C* *independent of* *N* *such that*

9 $$\left| (u,\phi )-(u,\phi )_{N}\right| \le CN^{-m}|u|_{H^{m;N}(\Omega )}\Vert \phi \Vert _{L^2(\Omega )},$$

*where* $(\cdot ,\cdot )$ *represents the continuous inner product in* $L^2(\Omega )$ *space and*

$$\begin{aligned} & (u,\phi )_{N}= \sum \limits _{\Vert {\mathbf{j}}\Vert \le N}u(\theta _{j_1},\theta _{j_2},\ldots ,\theta _{j_d}) \phi (\theta _{j_1},\theta _{j_2},\ldots ,\theta _{j_d}) \omega _{j_1}\omega _{j_2}\ldots \omega _{j_d}. \end{aligned}$$

*The seminorm is defined as*

$$\begin{aligned} & |u|_{H^{m;N}(\Omega )}=\left( \sum _{k=\min (m,N+1)}^{m} \sum _{i=1}^d\left\| \frac{\partial ^ku}{\partial x_i^k}\right\| _{L^2(\Omega )}^2\right) ^{\frac{1}{2}}. \end{aligned}$$

*Note that only pure derivatives in each spatial direction appear in this expression*.

From Fedotov (2004), we have the following result on the Lebesgue constant for the Lagrange interpolation polynomials associated with the Jacobi-Gauss points.

### **Lemma 2**

*Let* $\Vert I_N\Vert _{\infty } :=\max \nolimits _{{\mathbf{x}}\in \bar{\Omega }}\sum \nolimits _{\Vert {\mathbf{k}}\Vert \le N}|F_{k_1}(x_1)F_{k_2}(x_2)\cdots F_{k_d}(x_d)|$, *we have*

10 $$\begin{aligned} \Vert I_N\Vert _{\infty }=\left\{ \begin{array}{ll} \mathcal {O}\left( (\log N)^d\right) , &\quad if\; -1<\alpha ,\beta \le -\frac{1}{2},\\ \mathcal {O}\left( (N^{\max (\alpha ,\beta )+\frac{1}{2}})^d\right) , &{}\quad if\; -\frac{1}{2}<\alpha ,\beta<\frac{1}{d}-\frac{1}{2},\\ \mathcal {O}\left( (N^{\alpha +\frac{1}{2}})^d\right) , &{} \quad if\; -1<\beta \le -\frac{1}{2},\;-\frac{1}{2}<\alpha<\frac{1}{d}-\frac{1}{2},\\ \mathcal {O}\left( (N^{\beta +\frac{1}{2}})^d\right) , &{}\quad if\; -1<\alpha \le -\frac{1}{2},\;-\frac{1}{2}<\beta <\frac{1}{d}-\frac{1}{2}.\\ \end{array}\right. \end{aligned}$$

### **Lemma 3**

*Assume that* $u({\mathbf{x}})\in H_\omega ^m(\Omega )$ *for* $m>\frac{d}{2}$ *and denote* $(I_{N}u)({\mathbf{x}})$ *its interpolation polynomial associated with the multidimensional Jacobi Gauss points* $\{\tilde{{\mathbf{x}}}_{\mathbf{j}},\Vert {\mathbf{j}}\Vert \le N\}$. *Then the following estimates hold*

11 $$\Vert u-I_{N}u\Vert _{L_\omega ^2(\Omega )}\le CN^{-m}|u|_{H_\omega ^{m;N}(\Omega )},$$

12 $$\Vert u-I_{N}u\Vert _{L^\infty (\Omega )}\le CN^{d+2-m}|u|_{H_\omega ^{m;N}(\Omega )}.$$

### *Proof*

The inequality (11) can be found in Canuto et al. (2006). We now prove (12). From Canuto et al. (2006), we have

$$\Vert u-I_Nu\Vert _{H_\omega ^l(\Omega )}\le CN^{2l-m}|u|_{H_\omega ^{m;N}(\Omega )},\quad 0\le l\le m.$$

We know that $H_\omega ^l(\Omega )$ is embedded in $C(\bar{\Omega })$ for $l>\frac{d}{2}$, namely,

$$\begin{aligned} & \Vert u-I_Nu\Vert _{L^\infty (\Omega )}\le C\Vert u-I_Nu\Vert _{H_\omega ^l(\Omega )}\le CN^{2l-m}|u|_{H_\omega ^{m;N}(\Omega )} \\&\quad \le \left\{ \begin{array}{ll} CN^{d+2-m}|u|_{H_\omega ^{m;N}(\Omega )}, &{} \quad when\; d\; is\; an\; even\; number,\\ C N^{d+1-m}|u|_{H_\omega ^{m;N}(\Omega )}, &{}\quad when\; d\; is\; an\; odd \;number. \end{array}\right. \\&\quad \le CN^{d+2-m}|u|_{H_\omega ^{m;N}(\Omega )}. \end{aligned}$$

$$\square$$

The following Gronwall Lemma, whose proof can be found in Headley (1974), will be essential for establishing our main results.

### **Lemma 4**

*Suppose* $M\ge 0,$ *a nonnegative integrable function* $E({\mathbf{x}})$ *satisfies*

$$E(x_1,x_2,\ldots ,x_d)\le M\int _{-1}^{x_1}\int _{-1}^{x_2}\cdots \int _{-1}^{x_d}E(\tau _1,\tau _2,\ldots ,\tau _d)d\tau _d\ldots d\tau _2d\tau _1 +G(x_1,x_2,\ldots ,x_d),\quad (x_1,x_2,\ldots ,x_d)\in \Omega,$$

*where* $G({\mathbf{x}})$ *is also an integrable function, we have*

13 $$\Vert E\Vert _{L_\omega ^2(\Omega )}\le C\Vert G\Vert _{L_\omega ^2(\Omega )},$$

14 $$\Vert E\Vert _{L^\infty (\Omega )}\le C\Vert G\Vert _{L^\infty (\Omega )}.$$

From Theorem 1 in Nevai (1984), we have the following mean convergence result of Lagrange interpolation based at the multidimensional Jacobi-Gauss points.

### **Lemma 5**

*For every bounded function* $v({\mathbf{x}})$, *there exists a constant* *C* *independent of* *v* *such that*

15 $$\sup _N\left\| \sum \limits _{\Vert {\mathbf{j}}\Vert \le N}v(\tilde{{\mathbf{x}}}_{\mathbf{j}})F_{\mathbf{j}}({\mathbf{x}})\right\| _{L_\omega ^2(\Omega )}\le C\max _{{\mathbf{x}}\in \bar{\Omega }}|v({\mathbf{x}})|.$$

*For* $r\ge 0$ *and* $\kappa \in (0,1)$, ${\mathcal {C}}^{r,\kappa }(\bar{\Omega })$ *will denote the space of functions whose* *r*-*th derivatives are* $H{\ddot{o}}lder$ *continuous with exponent* $\kappa$, *endowed with the norm*:

$$\begin{aligned} & \Vert v\Vert _{C^{r,\kappa }(\bar{\Omega })}=\max \limits _{|\alpha |\le r} \max \limits _{{\mathbf{x}}\in \bar{\Omega }} \left| \frac{\partial ^{|\alpha |}v({\mathbf{x}})}{\partial x_1^{\alpha _1}\partial x_2^{\alpha _2}\cdots \partial x_d^{\alpha _d}}\right| \\&\quad +\max \limits _{|\alpha |\le r}\sup \limits _{{\mathbf{x}}^{'}\ne {\mathbf{x}}^{''}\in \bar{\Omega }} \left| \frac{\frac{\partial ^{|\alpha |}v({\mathbf{x}}^{'})}{\partial x_1^{\alpha _1}\partial x_2^{\alpha _2}\cdots \partial x_d^{\alpha _d}}-\frac{\partial ^{|\alpha |}v({\mathbf{x}}^{''})}{\partial x_1^{\alpha _1}\partial x_2^{\alpha _2}\cdots \partial x_d^{\alpha _d}}}{\left( (x_1^{'}-x_1^{''})^2+(x_2^{'}-x_2^{''})^2+ \cdots +(x_d^{'}-x_d^{''})^2\right) ^{\frac{\kappa }{2}}}\right| . \end{aligned}$$

*We shall make use of a result of * Ragozin (1970, (1971) *in the following lemma*.

### **Lemma 6**

*For nonnegative integer* *r* *and* $\kappa \in (0,1)$, *there exists a constant* $C_{r,\kappa }>0$ *such that for any function* $v\in {\mathcal {C}}^{r,\kappa }(\bar{\Omega })$, *there exists a polynomial function* ${\mathcal {T}}_Nv\in {\mathbb {P}}_N$ *such that*

16 $$\Vert v-{\mathcal {T}}_Nv\Vert _{L^\infty (\Omega )}\le C_{r,\kappa }N^{-(r+\kappa )}\Vert v\Vert _{{\mathcal {C}}^{r,\kappa }(\bar{\Omega })},$$

*Actually*, ${\mathcal {T}}_N$ *is a linear operator from* ${\mathcal {C}}^{r,\kappa }(\bar{\Omega })$ *into* ${\mathbb {P}}_N$.

### **Lemma 7**

*Assume there are constants* $L_0, L_1,L_2,\ldots ,L_d$ *such that*

$$\begin{aligned} & |\hat{K}(x_1,\tau _1,x_2,\tau _2,\ldots ,x_d,\tau _d, v_1)-\hat{K}(x_1,\tau _1,x_2,\tau _2,\ldots ,x_d,\tau _d, v_2)|\le L_0|v_1-v_2|,\\& |\hat{K}_{x_i}(x_1,\tau _1,x_2,\tau _2,\ldots ,x_d,\tau _d, v_1)-\hat{K}_{x_i}(x_1,\tau _1,x_2,\tau _2,\ldots ,x_d,\tau _d, v_2)|\le L_i|v_1-v_2|,\\&\quad i=1,2,\ldots ,d. \end{aligned}$$

*Let* $M_{v_1,v_2}$ *be defined by*

17 $$M_{v_1,v_2}({\mathbf{x}})=\int _{-1}^{x_1}\int _{-1}^{x_2}\cdots \int _{-1}^{x_d}[\hat{K}(x_1,\tau _1,x_2,\tau _2,\ldots ,x_d,\tau _d, v_1(\tau _1,\tau _2,\ldots ,\tau _d)) -\hat{K}(x_1,\tau _1,x_2,\tau _2,\ldots ,x_d,\tau _d, v_2(\tau _1,\tau _2,\ldots ,\tau _d))]d\tau _d\ldots d\tau _2d\tau _1.$$

Then, for any $\kappa \in (0,1)$ and $v_1,v_2\in {\mathcal {C}}(\bar{\Omega })$, there exists a positive constant $C\thicksim L_0, L_1,L_2,\ldots ,L_d$ such that

18 $$\frac{|M_{v_1,v_2}({\mathbf{x}}^\prime )- M_{v_1,v_2}({\mathbf{x}}^{\prime \prime })|}{\left( (x_1^{'}-x_1^{''})^2+(x_2^{'}-x_2^{''})^2+ \cdots +(x_d^{'}-x_d^{''})^2\right) ^{\frac{\kappa }{2}}} \le C\max _{{\mathbf{x}}\in \bar{\Omega }}|v_1({\mathbf{x}})-v_2({\mathbf{x}})|,$$

for any ${\mathbf{x}}^\prime , {\mathbf{x}}^{\prime \prime }\in \bar{\Omega }$ and ${\mathbf{x}}^\prime \ne {\mathbf{x}}^{\prime \prime }$. This implies that

19 $$\Vert M_{v_1,v_2}\Vert _{{\mathcal {C}}^{0,\kappa }(\bar{\Omega })}\le C\max _{{\mathbf{x}}\in \bar{\Omega }}|v_1({\mathbf{x}})-v_2({\mathbf{x}})|.$$

### *Proof*

For ease of exposition, and without essential loss of generality, we will proof this lemma for $d=2$ and assume $x_1^{\prime \prime }<x_1^\prime$, $x_2^{\prime \prime }<x_2^\prime$,

20 $$\begin{aligned} & |M_{v_1,v_2}({\mathbf{x}}^\prime )- M_{v_1,v_2}({\mathbf{x}}^{\prime \prime })| \\&\quad =\left|\int _{-1}^{x_1^\prime }\int _{-1}^{x_2^\prime } [\hat{K}(x_1^\prime ,\tau _1,x_2^\prime ,\tau _2, v_1(\tau _1,\tau _2))-\hat{K}(x_1^\prime ,\tau _1,x_2^\prime ,\tau _2, v_2(\tau _1,\tau _2))]d\tau _2d\tau _1\right. \\&\quad\quad\left. -\int _{-1}^{x_1^{\prime \prime }}\int _{-1}^{x_2^{\prime \prime }} [\hat{K}(x_1^{\prime \prime },\tau _1,x_2^{\prime \prime },\tau _2,v_1(\tau _1,\tau _2)) -\hat{K}(x_1^{\prime \prime },\tau _1,x_2^{\prime \prime },\tau _2, v_2(\tau _1,\tau _2))]d\tau _2d\tau _1\right| \\&\quad \le E_1+E_2. \end{aligned}$$

Here,

21 $$\begin{aligned} & E_1=\left|\int _{-1}^{x_1^\prime }\int _{-1}^{x_2^\prime } [\hat{K}(x_1^\prime ,\tau _1,x_2^\prime ,\tau _2, v_1(\tau _1,\tau _2))-\hat{K}(x_1^\prime ,\tau _1,x_2^\prime ,\tau _2, v_2(\tau _1,\tau _2))]d\tau _2d\tau _1\right. \\&\quad\quad \left.-\int _{-1}^{x_1^{\prime }}\int _{-1}^{x_2^{\prime \prime }} [\hat{K}(x_1^{\prime },\tau _1,x_2^{\prime },\tau _2,v_1(\tau _1,\tau _2)) -\hat{K}(x_1^{\prime },\tau _1,x_2^{\prime },\tau _2, v_2(\tau _1,\tau _2))]d\tau _2d\tau _1\right| \\&\quad \le \int _{-1}^{x_1^\prime }\int _{x_2^{\prime \prime }}^{x_2^\prime } |\hat{K}(x_1^\prime ,\tau _1,x_2^\prime ,\tau _2, v_1(\tau _1,\tau _2))-\hat{K}(x_1^\prime ,\tau _1,x_2^\prime ,\tau _2, v_2(\tau _1,\tau _2))|d\tau _2d\tau _1 \\&\quad \le CL_0\Vert v_1-v_2\Vert _{L^\infty (\Omega )}(x_2^\prime -x_2^{\prime \prime }) \\&\quad \le CL_0\Vert v_1-v_2\Vert _{L^\infty (\Omega )}(x_2^\prime -x_2^{\prime \prime })^{1+\kappa } (x_2^\prime -x_2^{\prime \prime })^{-\kappa } \\&\quad \le C\Vert v_1-v_2\Vert _{L^\infty (\Omega )}[(x_1^\prime -x_1^{\prime \prime })^2+ (x_2^\prime -x_2^{\prime \prime })^2]^{-\frac{\kappa }{2}}, \end{aligned}$$

22 $$\begin{aligned} E_2 &=\left|\int _{-1}^{x_1^\prime }\int _{-1}^{x_2^{\prime \prime }} [\hat{K}(x_1^\prime ,\tau _1,x_2^\prime ,\tau _2, v_1(\tau _1,\tau _2))-\hat{K}(x_1^\prime ,\tau _1,x_2^\prime ,\tau _2, v_2(\tau _1,\tau _2))]d\tau _2d\tau _1\right. \\&\quad\quad \left.-\int _{-1}^{x_1^{\prime \prime }}\int _{-1}^{x_2^{\prime \prime }} [\hat{K}(x_1^{\prime \prime },\tau _1,x_2^{\prime \prime },\tau _2,v_1(\tau _1,\tau _2)) -\hat{K}(x_1^{\prime \prime },\tau _1,x_2^{\prime \prime },\tau _2, v_2(\tau _1,\tau _2))]d\tau _2d\tau _1\right| \\ &\le \int _{-1}^{x_2^{\prime \prime }}(P_1+P_2+P_3)d\tau _2 \le C\max _{{\mathbf{x}}\in \bar{\Omega }}(P_1+P_2+P_3), \end{aligned}$$

where

23 $$\begin{aligned} & P_1=\left|\int _{-1}^{x_1^\prime } [\hat{K}(x_1^\prime ,\tau _1,x_2^\prime ,\tau _2, v_1(\tau _1,\tau _2))-\hat{K}(x_1^\prime ,\tau _1,x_2^\prime ,\tau _2, v_2(\tau _1,\tau _2))]d\tau _1\right. \\&\quad \left.-\int _{-1}^{x_1^{\prime }} [\hat{K}(x_1^{\prime },\tau _1,x_2^{\prime \prime },\tau _2,v_1(\tau _1,\tau _2)) -\hat{K}(x_1^{\prime },\tau _1,x_2^{\prime \prime },\tau _2, v_2(\tau _1,\tau _2))]d\tau _1\right| \\&\quad =\left|\int _{-1}^{x_1^\prime } [\hat{K}_{x_2}(x_1^\prime ,\tau _1,\xi ,\tau _2, v_1(\tau _1,\tau _2))-\hat{K}_{x_2}(x_1^\prime ,\tau _1,\xi ,\tau _2, v_2(\tau _1,\tau _2))](x_2^\prime -x_2^{\prime \prime })d\tau _1\right| \\&\quad \le CL_2\Vert v_1-v_2\Vert _{L^\infty (\Omega )}(x_2^\prime -x_2^{\prime \prime }) \\&\quad \le C\Vert v_1-v_2\Vert _{L^\infty (\Omega )}(x_2^\prime -x_2^{\prime \prime })^{1+\kappa } (x_2^\prime -x_2^{\prime \prime })^{-\kappa } \\&\quad \le C\Vert v_1-v_2\Vert _{L^\infty (\Omega )}[(x_1^\prime -x_1^{\prime \prime })^2+ (x_2^\prime -x_2^{\prime \prime })^2]^{-\frac{\kappa }{2}},\quad\quad \exists \,\xi \in (x_2^{\prime \prime },x_2^\prime ). \end{aligned}$$

similarly,

24 $$\begin{aligned} & P_2=\left|\int _{-1}^{x_1^\prime } [\hat{K}(x_1^\prime ,\tau _1,x_2^{\prime \prime },\tau _2, v_1(\tau _1,\tau _2))-\hat{K}(x_1^\prime ,\tau _1,x_2^{\prime \prime },\tau _2, v_2(\tau _1,\tau _2))]d\tau _1 \right.\\&\left.\quad\quad -\int _{-1}^{x_1^{\prime }} [\hat{K}(x_1^{\prime \prime },\tau _1,x_2^{\prime \prime },\tau _2,v_1(\tau _1,\tau _2)) -\hat{K}(x_1^{\prime \prime },\tau _1,x_2^{\prime \prime },\tau _2, v_2(\tau _1,\tau _2))]d\tau _1\right| \\&\quad =\left|\int _{-1}^{x_1^\prime } [\hat{K}_{x_1}(\eta ,\tau _1,x_2^{\prime \prime },\tau _2, v_1(\tau _1,\tau _2))-\hat{K}_{x_1}(\eta ,\tau _1,x_2^{\prime \prime },\tau _2, v_2(\tau _1,\tau _2))](x_1^\prime -x_1^{\prime \prime })d\tau _1\right| \\&\quad \le CL_1\Vert v_1-v_2\Vert _{L^\infty (\Omega )}(x_1^\prime -x_1^{\prime \prime }) \\&\quad \le C\Vert v_1-v_2\Vert _{L^\infty (\Omega )}[(x_1^\prime -x_1^{\prime \prime })^2+ (x_2^\prime -x_2^{\prime \prime })^2]^{-\frac{\kappa }{2}},\;\;\;\;\exists \;\eta \in (x_1^{\prime \prime },x_1^\prime ). \end{aligned}$$

25 $$\begin{aligned} & P_3=\left|\int _{-1}^{x_1^\prime } [\hat{K}(x_1^{\prime \prime },\tau _1,x_2^{\prime \prime },\tau _2, v_1(\tau _1,\tau _2))-\hat{K}(x_1^{\prime \prime },\tau _1,x_2^{\prime \prime },\tau _2, v_2(\tau _1,\tau _2))]d\tau _1 \right.\\&\quad\quad \left.-\int _{-1}^{x_1^{\prime \prime }} [\hat{K}(x_1^{\prime \prime },\tau _1,x_2^{\prime \prime },\tau _2,v_1(\tau _1,\tau _2)) -\hat{K}(x_1^{\prime \prime },\tau _1,x_2^{\prime \prime },\tau _2, v_2(\tau _1,\tau _2))]d\tau _1\right| \\&\quad \le \int _{x_1^{\prime \prime }}^{x_1^{\prime }}L_0|v_1-v_2|d\tau _1 \\&\quad \le C\Vert v_1-v_2\Vert _{L^\infty (\Omega )}(x_1^\prime -x_1^{\prime \prime })^{1+\kappa } (x_1^\prime -x_1^{\prime \prime })^{-\kappa } \\&\quad \le C\Vert v_1-v_2\Vert _{L^\infty (\Omega )}[(x_1^\prime -x_1^{\prime \prime })^2+ (x_2^\prime -x_2^{\prime \prime })^2]^{-\frac{\kappa }{2}}. \end{aligned}$$

The estimate (18) for $d = 2$ is obtained by combining (20)–(24). $\square$

## Error estimates

### **Theorem 1**

*Let* $u({\mathbf{x}})$ *be the exact solution of the multidimensional nonlinear Volterra integral equation* (3), *which is smooth*. $u_N({\mathbf{x}})$ *is the approximate solution, i.e*., $u({\mathbf{x}})\approx u_N({\mathbf{x}}).$ *Assume that*

$$\begin{aligned} & \left| \frac{\partial ^k}{\partial \theta _i^k}\tilde{K}(x_1,\theta _1, x_2,\theta _2,\ldots , x_d,\theta _d,v_1) -\frac{\partial ^k}{\partial \theta _i^k}\tilde{K}(x_1,\theta _1, x_2,\theta _2,\ldots , x_d,\theta _d,v_2)\right| \\&\le L_{ik}|v_1-v_2|,\quad i=1,2,\ldots ,d;\quad k=1,2,\ldots ,m,\\& L=\max \limits _{1\le i\le d,1\le k\le m}L_{ik}. \end{aligned}$$

*Then there is a constant* *C* *such that the errors satisfy for* $m>d+2$,

26 $$\begin{aligned} & ||u-u_N||_{L^\infty (\Omega )}\le CN^{-m} \\&\left\{ \begin{array}{ll} (\log N)^dK^{*}+N^{d+2}|u|_{H_\omega ^{m;N}(\Omega )}, &{}\quad if\; -1<\alpha ,\beta \le -\frac{1}{2},\\ \left( N^{\max (\alpha ,\beta )+\frac{1}{2}}\right) ^dK^{*}+N^{d+2}|u|_{H_\omega ^{m;N}(\Omega )}, &{} \quad if\; -\frac{1}{2}<\alpha ,\beta<\frac{1}{d}-\frac{1}{2},\\ \left( N^{\alpha +\frac{1}{2}}\right) ^dK^{*}+N^{d+2}|u|_{H_\omega ^{m;N}(\Omega )}, &{} \quad if\; -1<\beta \le -\frac{1}{2},\;-\frac{1}{2}<\alpha<\frac{1}{d}-\frac{1}{2},\\ \left( N^{\beta +\frac{1}{2}}\right) ^dK^{*}+N^{d+2}|u|_{H_\omega ^{m;N}(\Omega )}, &{} \quad if\; -1<\alpha \le -\frac{1}{2},\;-\frac{1}{2}<\beta <\frac{1}{d}-\frac{1}{2}.\\ \end{array}\right. \end{aligned}$$

*where*

$$\begin{aligned} & K^{*}=\max \limits _{\Vert {\mathbf{j}}\Vert \le N} |\tilde{K}({\tilde{x}}_{j_1},\theta _1, {\tilde{x}}_{j_2},\theta _2,\ldots , {\tilde{x}}_{j_d},\theta _d,u(\theta _1,\theta _2,\ldots ,\theta _d))|_{H^{m;N} (\Omega )},\\& C\thicksim d, L, L_0, L_1, L_2, \ldots , L_d. \end{aligned}$$

### *Proof*

We subtract (8) from (5) to get the error equation

$$u({\tilde{x}}_{j_1},{\tilde{x}}_{j_2},\ldots ,{\tilde{x}}_{j_d}) -u_{j_1j_2\ldots j_d} +\int _{-1}^1\int _{-1}^1\cdots \int _{-1}^1 [\tilde{K}({\tilde{x}}_{j_1},\tau _1({\tilde{x}}_{j_1},\theta _1), {\tilde{x}}_{j_2},\tau _2({\tilde{x}}_{j_2},\theta _2),\ldots , {\tilde{x}}_{j_d},\tau _d({\tilde{x}}_{j_d},\theta _d), u(\tau _1({\tilde{x}}_{j_1},\theta _1), \tau _2({\tilde{x}}_{j_2},\theta _2),\ldots ,\tau _d({\tilde{x}}_{j_d},\theta _d))) -\tilde{K}({\tilde{x}}_{j_1},\tau _1({\tilde{x}}_{j_1},\theta _1), {\tilde{x}}_{j_2},\tau _2({\tilde{x}}_{j_2},\theta _2),\ldots , {\tilde{x}}_{j_d},\tau _d({\tilde{x}}_{j_d},\theta _d), u_N(\tau _1({\tilde{x}}_{j_1},\theta _1), \tau _2({\tilde{x}}_{j_2},\theta _2),\ldots ,\tau _d({\tilde{x}}_{j_d},\theta _d)))]d\theta _d\cdots d\theta _2d\theta _1 =I({\tilde{x}}_{j_1},{\tilde{x}}_{j_2},\ldots ,{\tilde{x}}_{j_d}),$$

where

$$I({\tilde{x}}_{j_1},{\tilde{x}}_{j_2},\ldots ,{\tilde{x}}_{j_d}) =\sum \limits _{\Vert {\mathbf{k}}\Vert \le N} \tilde{K}({\tilde{x}}_{j_1},\tau _1({\tilde{x}}_{j_1},\theta _{k_1}), {\tilde{x}}_{j_2},\tau _2({\tilde{x}}_{j_2},\theta _{k_2}),\ldots , {\tilde{x}}_{j_d},\tau _d({\tilde{x}}_{j_d},\theta _{k_d}), u_N(\tau _1({\tilde{x}}_{j_1},\theta _{k_1}), \tau _2({\tilde{x}}_{j_2},\theta _{k_2}),\ldots ,\tau _d({\tilde{x}}_{j_d},\theta _{k_d}))) \omega _{k_1}\omega _{k_2}\ldots \omega _{k_d} -\int _{-1}^1\int _{-1}^1\cdots \int _{-1}^1 \tilde{K}({\tilde{x}}_{j_1},\tau _1({\tilde{x}}_{j_1},\theta _1), {\tilde{x}}_{j_2},\tau _2({\tilde{x}}_{j_2},\theta _2),\ldots , {\tilde{x}}_{j_d},\tau _d({\tilde{x}}_{j_d},\theta _d), u_N(\tau _1({\tilde{x}}_{j_1},\theta _1), \tau _2({\tilde{x}}_{j_2},\theta _2),\ldots ,\tau _d({\tilde{x}}_{j_d},\theta _d))) d\theta _d\ldots d\theta _2d\theta _1.$$

Using the variable transformation (6), we have

27 $$\begin{aligned} & u({\tilde{x}}_{j_1},{\tilde{x}}_{j_2},\ldots ,{\tilde{x}}_{j_d}) -u_{j_1j_2\ldots j_d} \\&\quad\quad +\int _{-1}^{{\tilde{x}}_{j_1}}\int _{-1}^{{\tilde{x}}_{j_2}} \ldots \int _{-1}^{{\tilde{x}}_{j_d}} [\hat{K}({\tilde{x}}_{j_1},\tau _1, {\tilde{x}}_{j_2},\tau _2,\ldots , {\tilde{x}}_{j_d},\tau _d,u(\tau _1, \tau _2,\ldots ,\tau _d)) \\&\quad\quad -\hat{K}({\tilde{x}}_{j_1},\tau _1, {\tilde{x}}_{j_2},\tau _2,\ldots , {\tilde{x}}_{j_d},\tau _d,u_N(\tau _1, \tau _2,\ldots ,\tau _d))]d\tau _d\ldots d\tau _2d\tau _1 \\&\quad =I({\tilde{x}}_{j_1},{\tilde{x}}_{j_2},\ldots ,{\tilde{x}}_{j_d}). \end{aligned}$$

Multiplying $F_{j_1}(x_1)F_{j_2}(x_2)\ldots F_{j_d}(x_d)$ on both sides of Eq. (27) and summing up $\Vert {\mathbf{j}}\Vert \le N$ yield

28 $$e_u(x_1, x_2,\ldots ,x_d)+ \int _{-1}^{x_1}\int _{-1}^{x_2} \cdots \int _{-1}^{x_d} [\hat{K}(x_1,\tau _1, x_2,\tau _2,\ldots , x_d,\tau _d,u(\tau _1, \tau _2,\ldots ,\tau _d)) -\hat{K}(x_1,\tau _1, x_2,\tau _2,\ldots , x_d,\tau _d,u_N(\tau _1, \tau _2,\ldots ,\tau _d))] d\tau _d\ldots d\tau _2d\tau _1 =J_1(x_1, x_2,\ldots ,x_d)+J_2(x_1, x_2,\ldots ,x_d)+J_3(x_1, x_2,\ldots ,x_d).$$

Consequently,

29 $$\begin{aligned} & |e_u(x_1, x_2,\ldots ,x_d)|\le L_0\int _{-1}^{x_1}\int _{-1}^{x_2} \ldots \int _{-1}^{x_d} |e_u(\tau _1, \tau _2,\ldots ,\tau _d)| d\tau _d\ldots d\tau _2d\tau _1 \\&\quad +|J_1(x_1, x_2,\ldots ,x_d)|+|J_2(x_1, x_2,\ldots ,x_d)|+|J_3(x_1, x_2,\ldots ,x_d)|, \end{aligned}$$

where

$$\begin{aligned} J_1({\mathbf{x}})&= \sum \limits _{\Vert {\mathbf{j}}\Vert \le N} I({\tilde{x}}_{j_1},{\tilde{x}}_{j_2},\ldots ,{\tilde{x}}_{j_d}) F_{j_1}(x_1)F_{j_2}(x_2)\ldots F_{j_d}(x_d), \\ J_2({\mathbf{x}})= & {} u(x_1, x_2,\ldots ,x_d)-(I_Nu)(x_1, x_2,\ldots ,x_d), \\ J_3({\mathbf{x}}) &= \int _{-1}^{x_1}\int _{-1}^{x_2} \cdots \int _{-1}^{x_d} [\hat{K}(x_1,\tau _1, x_2,\tau _2,\ldots , x_d,\tau _d,u(\tau _1, \tau _2,\ldots ,\tau _d)) \\&\quad -\hat{K}(x_1,\tau _1, x_2,\tau _2,\ldots , x_d,\tau _d,u_N(\tau _1, \tau _2,\ldots ,\tau _d))] d\tau _d\ldots d\tau _2d\tau _1 \\&\quad -I_N\int _{-1}^{x_1}\int _{-1}^{x_2} \cdots \int _{-1}^{x_d} [\hat{K}(x_1,\tau _1, x_2,\tau _2,\ldots , x_d,\tau _d,u(\tau _1, \tau _2,\ldots ,\tau _d)) \\&\quad -\hat{K}(x_1,\tau _1, x_2,\tau _2,\ldots , x_d,\tau _d,u_N(\tau _1, \tau _2,\ldots ,\tau _d))] d\tau _d\ldots d\tau _2d\tau _1. \end{aligned}$$

It follows from the Gronwall inequality in Lemma 4 that

30 $$\Vert e_u\Vert _{L_\infty (\Omega )}\le C\left( \Vert J_1\Vert _{L_\infty (\Omega )}+\Vert J_2\Vert _{L_\infty (\Omega )} +\Vert J_3\Vert _{L_\infty (\Omega )}\right) .$$

Using (9) and (10), we have

31 $$\begin{aligned} & ||J_1||_{L_\infty (\Omega )}\le C\Vert I_N\Vert _\infty \left( \max \limits _{\Vert {\mathbf{j}}\Vert \le N}|I({\tilde{x}}_{j_1},{\tilde{x}}_{j_2},\ldots ,{\tilde{x}}_{j_d})|\right) \\&\quad \le C\Vert I_N\Vert _\infty N^{-m}|\tilde{K}({\tilde{x}}_{j_1},\theta _1, {\tilde{x}}_{j_2},\theta _2,\ldots , {\tilde{x}}_{j_d},\theta _d,u_N(\theta _1, \theta _2,\ldots ,\theta _d))|_{H^{m;N}(\Omega )}, \\&\quad \le C\Vert I_N\Vert _\infty N^{-m}(K^*+|\tilde{K}({\tilde{x}}_{j_1},\theta _1, {\tilde{x}}_{j_2},\theta _2,\ldots , {\tilde{x}}_{j_d},\theta _d,u_N(\theta _1, \theta _2,\ldots ,\theta _d)) \\&\quad\quad -\tilde{K}({\tilde{x}}_{j_1},\theta _1, {\tilde{x}}_{j_2},\theta _2,\ldots , {\tilde{x}}_{j_d},\theta _d,u(\theta _1, \theta _2,\ldots ,\theta _d))|_{H^{m;N}(\Omega )}). \end{aligned}$$

A straightforward computation shows that

32 $$\begin{aligned} & |\tilde{K}({\tilde{x}}_{j_1},\theta _1, {\tilde{x}}_{j_2},\theta _2,\ldots , {\tilde{x}}_{j_d},\theta _d,u_N(\theta _1, \theta _2,\ldots ,\theta _d)) \\&\quad\quad -\tilde{K}({\tilde{x}}_{j_1},\theta _1, {\tilde{x}}_{j_2},\theta _2,\ldots , {\tilde{x}}_{j_d},\theta _d,u(\theta _1, \theta _2,\ldots ,\theta _d))|_{H^{m;N}(\Omega )} \\&\quad \le \left( \sum _{k=1}^m\sum _{i=1}^d\Vert \frac{\partial ^k}{\partial \theta _i^k}\tilde{K}({\tilde{x}}_{j_1},\theta _1, {\tilde{x}}_{j_2},\theta _2,\ldots , {\tilde{x}}_{j_d},\theta _d,u_N(\theta _1, \theta _2,\ldots ,\theta _d))\right. \\&\quad\quad \left. -\frac{\partial ^k}{\partial \theta _i^k}\tilde{K}({\tilde{x}}_{j_1},\theta _1, {\tilde{x}}_{j_2},\theta _2,\ldots , {\tilde{x}}_{j_d},\theta _d,u(\theta _1, \theta _2,\ldots ,\theta _d))\Vert _{L^2(\Omega )}^2\right) ^{\frac{1}{2}} \\&\quad \le \left( \sum _{k=1}^m\sum _{i=1}^dL_{ik}\Vert u_N-u\Vert _{L^2(\Omega )}^2\right) ^{\frac{1}{2}} \\&\quad \le L^{\frac{m\times d}{2}}\Vert u_N-u\Vert _{L^2(\Omega )}\le C\Vert e_u\Vert _{L^\infty (\Omega )}. \end{aligned}$$

Due to Lemma 3,

33 $$\Vert J_2\Vert _{L^\infty (\Omega )}\le CN^{d+2-m}|u|_{H_\omega ^{m;N}(\Omega )}.$$

By virtue of Lemmas 6 and 7,

34 $$\begin{aligned} & \Vert J_3\Vert _{L^\infty (\Omega )} =\Vert (I-I_N)M_{u,u_N}\Vert _{L^\infty (\Omega )} \\&\quad =\Vert (I-I_N)(M_{u,u_N}-{\mathcal {T}}_NM_{u,u_N})\Vert _{L^\infty (\Omega )} \\&\quad \le (1+\Vert I_N\Vert _\infty )\Vert M_{u,u_N}-{\mathcal {T}}_NM_{u,u_N}\Vert _{L^\infty (\Omega )} \\&\quad \le C\Vert I_N\Vert _\infty N^{-\kappa }\Vert M_{u,u_N}\Vert _{C^{0,\kappa }(\bar{\Omega })} \\&\quad \le C\Vert I_N\Vert _\infty N^{-\kappa }\Vert e_u\Vert _{L^\infty (\Omega )} \\&\quad \left\{ \begin{array}{ll} C(\log N)^dN^{-\kappa }\Vert e_u\Vert _{L^\infty (\Omega )}, &{} \quad if\; -1<\alpha ,\beta \le -\frac{1}{2},\\ C(N^{\max (\alpha ,\beta )+\frac{1}{2}})^dN^{-\kappa }\Vert e_u\Vert _{L^\infty (\Omega )}, &{} \quad if\; -\frac{1}{2}<\alpha ,\beta<\frac{1}{d}-\frac{1}{2},\\ C(N^{\alpha +\frac{1}{2}})^dN^{-\kappa }\Vert e_u\Vert _{L^\infty (\Omega )}, &{}\quad if\; -1<\beta \le -\frac{1}{2},\;-\frac{1}{2}<\alpha<\frac{1}{d}-\frac{1}{2},\\ C(N^{\beta +\frac{1}{2}})^dN^{-\kappa }\Vert e_u\Vert _{L^\infty (\Omega )}, &{} \quad if\; -1<\alpha \le -\frac{1}{2},\;-\frac{1}{2}<\beta <\frac{1}{d}-\frac{1}{2}.\\ \end{array}\right. \end{aligned}$$

We now obtain the estimate for $\Vert e_{u}\Vert _{L^\infty (\Omega )}$ by using (30)–(34),

$$\Vert e_{u}\Vert _{L^\infty (\Omega )}\le CN^{-m} \left( \Vert I_N\Vert _\infty K^{*}+N^{d+2}|u|_{H_\omega ^{m;N}(\Omega )} \right) ,$$

where in last step we have used the following assumption,

35 $$\begin{aligned} & \left\{ \begin{array}{ll} 0<\kappa<1, &{}\quad if\; -1<\alpha ,\beta \le -\frac{1}{2},\\ (\max (\alpha ,\beta )+\frac{1}{2})^d<\kappa<1, &{}\quad if\; -\frac{1}{2}<\alpha ,\beta<\frac{1}{d}-\frac{1}{2},\\ (\alpha +\frac{1}{2})^d<\kappa<1, &{}\quad if\; -1<\beta \le -\frac{1}{2},\;-\frac{1}{2}<\alpha<\frac{1}{d}-\frac{1}{2},\\ (\beta +\frac{1}{2})^d<\kappa<1, &{}\quad if\; -1<\alpha \le -\frac{1}{2},\;-\frac{1}{2}<\beta <\frac{1}{d}-\frac{1}{2}.\\ \end{array}\right. \end{aligned}$$

This completes the proof of the theorem. $\square$

### **Theorem 2**

*If the hypotheses given in Theorem* 1 *hold and* $\kappa$ *satisfies* (35), *then*

36 $$\begin{aligned} & \Vert u-u_N\Vert _{L_\omega ^2(\Omega )}\le CN^{-m} \\&\quad \left\{ \begin{array}{ll} (1+N^{-\kappa }(\log N)^d)K^* +(1+N^{d+2-\kappa }) |u|_{H_\omega ^{m;N}(\Omega )},&{} \\ \quad \quad\quad \quad\quad if\; -1<\alpha ,\beta \le -\frac{1}{2},\\ \left( 1+N^{-\kappa }\left( N^{\max (\alpha ,\beta )+\frac{1}{2}}\right) ^d\right) K^* +(1+N^{d+2-\kappa }) |u|_{H_\omega ^{m;N}(\Omega )},&{} \\ \quad\quad \quad\quad \quad if\; -\frac{1}{2}<\alpha ,\beta<\frac{1}{d}-\frac{1}{2},\\ (1+N^{-\kappa }\left( N^{\alpha +\frac{1}{2}})^d\right) K^* +(1+N^{d+2-\kappa }) |u|_{H_\omega ^{m;N}(\Omega )},&{} \\ \quad\quad \quad\quad \quad if\; -1<\beta \le -\frac{1}{2},\;-\frac{1}{2}<\alpha<\frac{1}{d}-\frac{1}{2},\\ \left( 1+N^{-\kappa }\left( N^{\beta +\frac{1}{2}}\right) ^d\right) K^* +(1+N^{d+2-\kappa }) |u|_{H_\omega ^{m;N}(\Omega )},&{} \\ \quad\quad \quad\quad \quad if\; -1<\alpha \le -\frac{1}{2},\;-\frac{1}{2}<\beta <\frac{1}{d}-\frac{1}{2}.\\ \end{array}\right. \end{aligned}$$

### *Proof*

By using (28) and Gronwall inequality in Lemma 4, we obtain that

37 $$\Vert e_{u}\Vert _{L_\omega ^2(\Omega )}\le C\left( \Vert J_1\Vert _{L_\omega ^2(\Omega )}+\Vert J_2\Vert _{L_\omega ^2(\Omega )}+\Vert J_3\Vert _{L_\omega ^2(\Omega )}\right) .$$

Using Lemmas 1, 5 and (32) we have for

38 $$\Vert J_1\Vert _{L_\omega ^2(\Omega )}\le C\max \limits _{{\mathbf{x}}\in \bar{\Omega }}|I({\mathbf{x}})| \le CN^{-m}(K^* +\Vert e_u\Vert _{L_\omega ^2(\Omega )}).$$

Due to Lemma 3,

39 $$\Vert J_2\Vert _{L_\omega ^2(\Omega )}\le CN^{-m}|u|_{H_\omega ^{m;N}(\Omega )}.$$

By virtue of Lemmas 6 and 7,

40 $$\begin{aligned} & \Vert J_3\Vert _{L_\omega ^2(\Omega )} =\Vert (I-I_N)M_{u,u_N}\Vert _{L_\omega ^2(\Omega )} \\&\quad =\Vert (I-I_N)(M_{u,u_N}-{\mathcal {T}}_NM_{u,u_N})\Vert _{L_\omega ^2(\Omega )} \\&\quad \le \Vert M_{u,u_N}-{\mathcal {T}}_NM_{u,u_N}\Vert _{L_\omega ^2(\Omega )}+\Vert I_N(M_{u,u_N}-{\mathcal {T}}_NM_{u,u_N})\Vert _{L_\omega ^2(\Omega )} \\&\quad \le C\Vert M_{u,u_N}-{\mathcal {T}}_NM_{u,u_N}\Vert _{L^\infty (\Omega )} \\&\quad \le CN^{-\kappa }\Vert e_u\Vert _{L^\infty (\Omega )} \\&\quad \le CN^{-m-\kappa } \left( \Vert I_N\Vert _{\infty }K^{*}+N^{d+2}|u|_{H_\omega ^{m;N}(\Omega )} \right) . \end{aligned}$$

The desired estimate (36) is obtained by combining (37)–(40) and using the same technique as in the proof of Theorem 1. $\square$

## Numerical results

We give two numerical examples to confirm our analysis. To examine the accuracy of the results, $L_\omega ^2$ and $L^\infty$ errors are employed to assess the efficiency of the method. All the calculations are supported by the software Matlab.

### *Example 1*

We consider the following two-dimensional Volterra integral equation

41 $$u(x,y)+\int _{-1}^x\int _{-1}^y \cos (x+y)e^{\frac{\xi \eta }{2}}u(\xi ,\eta )d\eta d\xi =e^{-\frac{xy}{2}}+\cos (x+y)(x+1)(y+1).$$

**Fig. 1:**
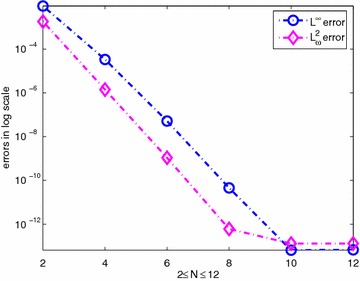
The errors $u-u_N$ versus the number of collocation points in $L^\infty$ and $L^2_{\omega }$ norms

The corresponding exact solution is given by $u(x,y)=e^{-\frac{xy}{2}}$. We select $\alpha =-\frac{2}{3},\;\beta =-\frac{1}{2}$. Table 1 shows the errors $\Vert u-u_N\Vert _{L_\omega ^2(\Omega )}$ and $\Vert u-u_N\Vert _{L^\infty (\Omega )}$ obtained by using the spectral collocation method described above. Furthermore, the numerical results are plotted for $2\le N\le 12$ in Fig. 1. It is observed that the desired exponential rate of convergence is obtained.

**Table 1:** The errors $\Vert u-u_N\Vert _{L_\omega ^2(\Omega )}$ and $\Vert u-u_N\Vert _{L^\infty (\Omega )}$

*N*	2	4	6
$L^\infty$-error	9.3273e−003	3.3409e−005	5.1698e−008
$L_\omega ^2$-error	1.8151e−003	1.4154e−006	1.0899e−009

*N*	8	10	12
$L^\infty$-error	4.5534e−011	6.5281e−014	6.7390e−014
$L_\omega ^2$-error	6.0859e−013	1.3022e−013	1.3124e−013

### *Example 2*

Consider the equation with

42 $$\begin{aligned} & v(x,y)+\int _{-1}^x\int _{-1}^ycos(x+\xi )v(\xi ,\eta )d\eta d\xi \\&\quad =sin(x+y)-\frac{1}{4}sin(3x+y)+\frac{1}{4}sin(x+y-2)-\frac{1}{2}(x+1)cos(x-y) \\&\quad\quad +\frac{1}{2}(x+1)cos(x+1)+\frac{1}{4}sin(3x-1)-\frac{1}{4}sin(x-3). \end{aligned}$$

**Fig. 2:**
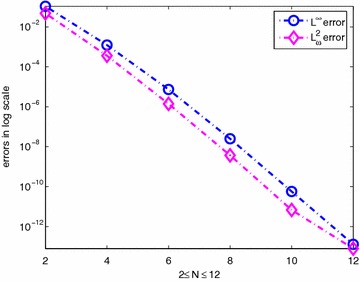
The errors $v-v_N$ versus the number of collocation points in $L^\infty$ and $L^2_{\omega }$ norms

The corresponding exact solution is given by $v(x,y)=sin(x+y)$. We select $\alpha =-\frac{2}{3},\;\beta =-\frac{3}{4}$. Table 2 shows the errors $\Vert v-v_N\Vert _{L_\omega ^2(\Omega )}$ and $\Vert v-v_N\Vert _{L^\infty (\Omega )}$. The numerical results are plotted for $2\le N\le 12$ in Fig. 2.

**Table 2:** The errors $\Vert v-v_N\Vert _{L_\omega ^2(\Omega )}$ and $\Vert v-v_N\Vert _{L^\infty (\Omega )}$

*N*	2	4	6
$L^\infty$-error	1.0746e−001	1.2307e−003	7.3430e−006
$L_\omega ^2$-error	4.7972e−002	3.5805e−004	1.3925e−006

*N*	8	10	12
$L^\infty$-error	2.5031e−008	5.6992e−011	1.2992e−013
$L_\omega ^2$-error	3.6864e−009	6.7815e−012	7.9865e−014

## Conclusions

In this paper, we proposed a spectral collocation method based on Jacobi orthogonal polynomials to obtain approximate solution for multidimensional nonlinear Volterra integral equation. The most important contribution of this work is that we are able to demonstrate rigorously that the errors of spectral approximations decay exponentially in both $L^\infty (\Omega )$ norm and $L^2_{\omega }(\Omega )$ norm on d-dimensional space, which is a desired feature for a spectral method.
